# Cross-Linked Carboxymethylcellulose Adsorbtion Membranes from *Ziziphus lotus* for the Removal of Organic Dye Pollutants

**DOI:** 10.3390/ma15248760

**Published:** 2022-12-08

**Authors:** Sara Saad, Izaskun Dávila, Amaia Morales, Jalel Labidi, Younes Moussaoui

**Affiliations:** 1 Laboratory for the Application of Materials to the Environment, Water and Energy (LR21ES15), Faculty of Sciences of Gafsa, University of Gafsa, Gafsa 2112, Tunisia; 2 Department of Chemical and Environmental Engineering, University of the Basque Country, UPV/EHU Plaza Europa 1, 20018 San Sebastián, Spain; 3 Faculty of Sciences of Gafsa, University of Gafsa, Gafsa 2112, Tunisia; 4 Department of Chemical and Environmental Engineering, University of the Basque Country, UPV/EHU Calle Nieves Cano 12, 01006 Vitoria-Gasteiz, Spain; 5 Organic Chemistry Laboratory (LR17ES08), Faculty of Sciences of Sfax, University of Sfax, Sfax 3029, Tunisia

**Keywords:** *Ziziphus lotus*, valorization, carboxymethylcellulose, eco-friendly adsorption membrane, methyl green

## Abstract

The goal of this study is to assess *Ziziphus lotus*’s potential for producing carboxymethylcellulose adsorption membranes with the ability to adsorb methyl green from wastewaters by the revalorization of its cellulosic fraction. The cellulose from this feedstock was extracted by an alkaline process and TAPPI standard technique T 203 cm-99 and afterwards they were carboxymethylated. The obtained carboxymethylcelluloses were deeply characterized, being observed that the carboxymethylcellulose produced from the alkaline cellulose presented the higher solubility due to its lower crystallinity degree (53.31 vs. 59.4%) and its higher substitution degree (0.85 vs. 0.74). This carboxymethylcellulose was cross-linked with citric acid in an aqueous treatment in order to form an adsorption membrane. The citric acid provided rigidity to the membrane and although it was hydrophilic it was not soluble in water. By evaluating the potential of the produced membrane for the removal of pollutant dyes from wastewater, it was observed that the adsorption membrane prepared from the carboxymethylcellulose’s produced from the *Ziziphus lotus* was able to remove 99% of the dye, methyl green, present in the wastewater. Thus, this work demonstrates the potential of the *Ziziphus lotus* for the production of a novel and cost-effective carboxymethylcellulose adsorption membrane with high capacity to treat wastewaters.

## 1. Introduction

The Earth is known as the blue planet due to its high water content, which occupies 71% of the Earth´s surface (1.38 × 10^9^ Km^3^). However, only 3% of the water present in the Earth is sweet water from which only 0.3% is available in lakes, swamps and rivers, among other resources [1]. The demographic increase that is expected for the following 30 years [2], the climate change and the unequal distribution of this resource could accrue the risk of water shortage in some arid and semi-arid regions [3,4]. To avoid the depletion of this essential resource and its overconsumption in domestic, agricultural and industrial uses, the recycling of the wastewaters generated by these activities have been evaluated as one of multiple potential solutions [5]. Among the different pollutants that could be contained in these wastes, organic compounds, such as dyes are one of the most important ones. Annually, approximately 1,000,000 tons of dyes are consumed worldwide by tanneries and by the food, cosmetic, textile, and medicinal sectors from which 50% is discharged to water [6]. Due to their synthetic origin and complex structure they present a high stability a low biodegradability, and they tend to accumulate on the surface of the water [7,8]. As a result, less sunlight can penetrate the water, increasing its turbidity, which could produce the death of aquatic life due to the low oxygen released into the water by the photosynthesis of the algae. Furthermore, they accumulate in the living cells of the fishes, so when they are inherited by humans they could cause depression of the immune system, allergic reactions, blade cancer or hyperactivity in children [9].

Some of the most employed procedures to remove dyes from wastewaters are coagulation and flocculation, foam flotation, membrane filtration or chemical processes. However, these treatments produce an accumulation of the pollutant in a solid phase, which does not solve the contamination problem since they can lixiviate again [10]. To solve this problem, there are more advanced wastewater treatment procedures that consist of chemical oxidation, ion exchange, electrochemical methods, or adsorption [10,11,12,13]. Adsorption is one of the therapies that is often used to remove dyes because of its simple design, simple operation, and relatively simple renewal of the adsorbent [12,13,14]. A wide range of adsorbents of a different nature could be employed to remove these pollutants from wastewater, for instance clays, zeolites, activated alumina, activated carbon, sludge, biomass derived polymers or industrial by-products [15,16]. Some of these adsorbents, such as the activated carbon, are expensive. Thus, in the latest years, the production of adsorbents derived from biomass has received great interest due to the high availability and low cost of these starting materials and because it permits the revalorization of agricultural or forestry residues [17,18].

In this context, the purpose of this study is to valorize the *Ziziphus lotus* by the production of a membrane with an adsorption capacity able to remove dyes from wastewaters. This feedstock is a bush from the *Rhamnaceae* family that is found abundantly in tropical and subtropical areas [19,20]. The fruit and leaves of the Ziziphus lotus are widely used in traditional medicine to treat several diseases, including bronchitis, diarrhea, abscess and diabetes due to the anti-inflammatory, analgesic, antibacterial, and antioxidant activities of their extracts. Apart from the medical applications, these feedstocks have also been employed for the obtaining of polysaccharides, antioxidants, or demmarane saponins [21,22]. Except for the fruit and leaves, the employment of the remaining part of this bush is scarce. Nevertheless, in the latest years, the Ziziphus lotus was subjected to a biorefinery process for the obtaining of oligosaccharides, lignin and cellulose nanofibers [23]. In this work, one of the main fractions of this feedstock, the cellulose, was employed for the production of adsorption membranes that could remove harmful dyes and heavy metal ions from wastewaters [24,25]. The cellulose due to its characteristic structure a neutral polymer and insoluble in water and in the most commonly employed solvent, so it could not be used to remove cationic dyes such as the methyl green, methylene blue or methyl orange, from wastewaters [17,26]. However, one of most commonly employed cellulose derivatives, the carboxymethylcellulose (CMC), could be employed to remove cationic dyes, since it is an anionic polysaccharide due to its multiple hydroxyl and carboxyl groups [26]. This polysaccharide has a great interest for the industry, since it is non-toxic, soluble in water, biodegradable and biocompatible [27,28]. Nevertheless, due to the solubility of the CMC, it is necessary to cross-link it with an organic acid, such as citric acid, in order to obtain a CMC membrane with the capacity to absorb cationic dyes [28]. Citric acid is a natural and, therefore, environmentally friendly and safe compound that has been used either for crosslinking cellulose nanofibers or carboxymethyl cellulose [28,29].

In this context, the present work evaluates the influence of two commonly employed cellulose extraction procedures (an alkaline process and a delignification-bleaching treatment based on a TAPPI (Technical Association of the Pulp and Paper Industry) standard method (T 203 cm-99)) [23,30] on the characteristics of the obtained CMC. The CMC with the most adequate properties will be cross-linked with citric acid to produce an eco-friendly adsorption membrane. To observe the effect caused by the cross-linking and to evaluate the potential benefits of producing adsorption membranes from an unexploited feedstock, such as the *Ziziphus lotus*, they were deeply characterized and their capacity to remove dyes, such as, methyl green (MG), was evaluated. Thus, the obtaining of adsorption membranes from unexploited biomass, such as, the *Ziziphus lotus* would permit the valorization of an under-valuated product and it would also improve or facilitate water remediation processes.

## 2. Materials and Methods

### 2.1. Raw Materials

The trunk of *Ziziphus lotus* was collected in Gafsa (Southwest of Tunisia). This biomass was composed by 30.8 ± 0.5 wt.% of glucan, 20.7 wt.% of hemicellulose, 19.6 ± 0.5 wt.% of Klason lignin and 1.49 wt.% of ash, as it was previously analyzed [23]. Prior to the extraction of the cellulose, the obtained feedstock was air-dried, milled and sieved to obtain particle size smaller than 5 mm. The milled feedstock was stored in a single lot in a dry place until further use.

### 2.2. Extraction and Bleaching of Alkaline Cellulose (PA-Cell)

The alkaline extraction of cellulose from the *Ziziphus lotus* chips was carried out by mixing the chips with a solution of 10% (*w*/*v*) of NaOH using a liquid/solid ratio of 1/10 (*g*/*g*) for 6 h at 80 °C [31]. Once the reactor was cooled down, the delignified chips were recovered by filtration and they were extensively cleaned with distilled water until neutral pH was achieved. Afterwards, they were bleached using 1.5 mL of NaClO_2_ and 0.5 mL of CH_3_COOH for 1 h at 70 °C [32]. To determine the yield of the treatment, the obtained PA-Cell was oven-dried at 100 °C for 24 h and weighted. This extraction and bleaching treatment was carried out in duplicates.

### 2.3. Extraction and Bleaching of α-Cellulose (α-Cell)

α-Cellulose was obtained according to TAPPI Standard method T 203 cm-99 [23]. This method consists of two steps, in the first step, *Ziziphus lotus* chips were mixed with NaClO_2_ using a solid/liquid ratio of 0.9% (*w*/*v*) and 0.5 mL of CH_3_COOH were added to adjust pH 3–4. The obtained mixture was kept for 1 h at 70 °C. Afterwards, two sequential doses of NaClO_2_, in a solid/liquid ratio of 0.9% (*w*/*v*), and of 0.5 mL of acetic acid were added to the reaction mixture. In the second step, the resulting solid residue was separated by filtration and it was rinsed with distilled water until the pH was neutralized. This solid was then exposed to 25 mL of a solution of 17.5% (*w*/*v*) NaOH solution for 30 min at 25 °C. To this mixture, 33 mL of distilled water was added and left for an additional 30 min. The resulting suspension was then filtered, rinsed with water until it reached neutrality, and stirred for 30 min. To determine the yield of the extraction and bleaching process the resulting α-Cellulose was oven-dried at 100 °C for 24 h and weighed. The obtained α-cellulose was stored at 4 °C until further use. The whole treatment was carried out in duplicates.

### 2.4. Carboxymethylation

The alkaline cellulose and the α-cellulose extracted from the trunk of the *Ziziphus lotus* were employed to produce carboxymethylcellulose (PA-CMC and α-CMC) by the procedure described by Heidrich and Ullmann [33]. Briefly, 2 g of each extracted fibers were mixed with 53 mL isopropanol under vigorous stirring. While the mixture was stirring, 10 mL of an aqueous solution of 40% NaOH was added over 20 min at room temperature. Afterwards, 2.4 g of monochloroacetic acid dissolved in 5 mL isopropanol were added for 20 min to the mixture and in order to conduct the etherification reaction it was kept at 40 °C for 1 h. Finally, the temperature of the mixture was increased to 70 °C and it was maintained for 4 h. Once the mixture was cooled down, it was filtrated and the obtained solid fraction was washed with methanol and neutralized with acetic acid. To clean the obtained solid and to remove undesirable by-product, it was washed three times with 70% (*v*/*v*) ethanol and then dried at 110 °C in an oven [33]. This process was carried out in duplicates.

### 2.5. Preparation of PA-CMC Adsorption Membrane

To elaborate the cross-linked adsorption membrane from the PA-CMC, 100 mL of a solution containing 20% (*w*/*v*) PA-CMC was prepared by dissolving the PA-CMC powder in deionized water at room temperature. Once the PA-CMC was dissolved, citric acid was added to the mixture in order to have a concentration of 20% (wt. cross-linking agent/wt. PA-CMC). The mixture was homogenized during 20 min, and then 10 mL of the mixture were placed onto a polystyrene Petri dish with a diameter of 60 mm. Then, the mixture was dried at 40 °C for 24 h to remove any remaining water. Finally, the samples were maintained at 80 °C for 24 h to carry out the cross-linking reaction.

### 2.6. Characterization Techniques

#### 2.6.1. Fiber Length Measurements and Degree of Polymerization (DPv)

To examine fiber lengths of the extracted cellulose, an aliquot of the PA-Cell and α-Cell were suspended in distilled water and passed through a MorFi analyzer (LB01, developed by Techpap-France and the Paper Technical Centre (Grenoble, France)). Dynamic light scattering (DLS) was used to determine the average size distribution of the produced CMC fibers (0.1% wt). The determination was carried out in a VASCO-2 particle size analyser (Nanosizer Cordouan Technologies, Pessac, France) using a measuring range of 2 to 6000 nm. Furthermore, the degree of polymerization (DPv) of the two produced carboxymethylcelluloses was measured as stated by to the NFT 12-005 standard, which consisted in the determination of the viscosity of samples of the fibrous fractions in a solution of cupriethylenediamine (CED). The degree of polymerization was estimated by the following Equation (1) [34]:

(1) $$\mathrm{DPv}=[0.75{\left(954{\mathrm{Log}}_{10}\eta -325\right)}^{1.105}$$

where η is the intrinsic viscosity (mPas).

All analyses were performed by duplicate.

#### 2.6.2. Determination of the Purity of the Carboxymethylcelluloses

To determine the purity of the CMC, 0.5 g of the CMC obtained from the PA-Cell and α-Cell were dissolved in 10 mL of distilled water and 10 mL of 1 M HCl was added to achieve the complete dissolution of the caboxymethylcellulose fibres. Once the fibers were dissolved five drops of phenolphthalein were added to the mixture and a solution of 1 M NaOH was added until the equivalence point was achieved. Afterwards, ethanol (50 mL, 95% (*v*/*v*)) was added slowly to the mixture under stirring. After that, 100 mL of ethanol (95% (*v*/*v*)) was added and the mixture was left standing for 15 min. Once the solution was settled, the supernatant liquid was removed by filtration and discarded. The obtained precipitate was washed four times with ethanol (80% (*v*/*v*)) and then washed again with 50 mL of ethanol (95% (*v*/*v*)). Finally, the precipitate was dried in an oven at 105 °C for 4 h.

#### 2.6.3. Determination of Degree of Substitution

The degree of substitution (DS) of the obtained PA-CMC and α-CMC was evaluated through acidometric titration. Briefly, 0.2 g of the CMC-s was dissolved in 50 mL of distilled water. Then, the pH of the solution was adjusted to pH 8 by the addition of NaOH. Finally, the solution was titrated with 0.05 M H_2_SO_4_ until a pH of 3.74 is achieved. The DS was estimated by using the formula that Dacrory et al. [35] detailed in their study:

(2) $$\mathrm{DS}=\frac{0.132\times B}{\left(1-0.08\right)\times B}$$

(3) $$B=2\times M\times \frac{V}{A}\times m$$

(4) $$A=\frac{m_{0}}{m}$$

where M is the molarity of H_2_SO_4_, V is the volume of H_2_SO_4_ used for titration, and B is the mmol/g of H_2_SO_4_ consumed per gram of material, A is the purity of the CMC, m_0_ and m are the weight of the purified carboxymethylated products before and after the purification.

#### 2.6.4. X-ray Diffraction (XRD) Analyses

XRD data were obtained using an X-ray diffractometer (D8-Advance Bruker AXS GmbH, Billerica, MA, USA) at room temperature with a monochromatic CuKα radiation in step-scan mode with a 2θ angle (from 10 to 45°) with a current of 4 mA and a scanning time of 5 min. To determine the crystallinity index (CrI) of the CMC through X-ray diffraction experiments, the method described by Segal et al. [36] was used. According to this empirical method, the CrI is calculated by the following Equation (5):

(5) $$\mathrm{CrI}\left(\%\right)=\frac{I_{200}-I_{\mathrm{am}}}{I_{200}}\cdot 100$$

where I_am_ is the intensity relative to the amorphous phase (18°) and I_200_ is the height of the maximum interference.

#### 2.6.5. Scanning Electron Microscope (SEM) Analyses

SEM is a technique that allows for the analysis of the morphology of the products by determining its structure, texture, and porosity. A ZEISS-ULTRA55 SEM microscope (Zeiss, Jena, Germany) was used to capture the micrographs of the untreated Ziziphus lotus, extracted alkaline cellulose, PA-CMC and the produced adsorption membrane.

#### 2.6.6. Fourier Transform Infrared (FTIR) Spectroscopy Analyses

FTIR analyses were performed using a PerkinElmer Spectrum Two FT-IR spectrometer (PerkinElmer, Waltham, MA, USA). The analyses of the extracted alkaline cellulose, the produced carboxymethylcellulose and PA-CMC adsorption membrane were carried out from 400 to 4000 cm^−1^ using a spectral resolution of 4 cm^−1^. 8 scans were recorded for each sample.

#### 2.6.7. Water Solubility Determination

To evaluate the effect caused by the cross-linking between the PA-CMC and the citric acid, the carboxymethylcelluloses obtained from the PA-Cell and the produced adsorption membrane were subjected to water solubility analyses. A rectangle of 1.5 × 3 cm^2^ of each sample were cut, dried at 106 °C for 3 h and weighed. Afterwards, the dried sample was introduced in 50 mL of distilled water and it was kept 24 h at room temperature under stirring. After 24 h, the remaining solid was dried at 106 °C for 24 h and weighted.

The water solubility was determined using the following Equation (6) [37]:

(6) $$\mathrm{WS}\;\left(\%\right)=\frac{\left(W_{0}-W_{1}\right)}{W_{0}}\cdot 100$$

where W_0_ is the weight of the rectangle of 1.5 × 3 cm^2^ (g) and W_1_ is the weight of the solid remaining after the water solubility analysis (g).

The water solubility determination was carried out in triplicate for each sample.

#### 2.6.8. Contact Angle Analyses

In order to determine the hydrophobicity or hydrophility of the PA-CMC adsorption membrane, it was subjected to a contact angle analysis using an Oca20 DataPhysiscs equipment (DataPhysics Instruments GmbH, Filderstadt, Germany). To carry out the analyses, a drop of 5 μL of distilled water was place on the surface of the adsorption membrane and the contact angle on the surface was determined at room temperature at 0, 5 and 10 s by the SCA20 software. The effect of each contact time was determined in triplicates [38].

#### 2.6.9. Mechanical Properties and Thermogravimetric Analyses (TGA)

To determine the effect caused by the cross-linking between the PA-CMC and the citric acid, the PA-CMC and the adsorption membrane produced form this carboxymethylcellulose were subjected to mechanical properties and TGA analyses.

The mechanical properties of both samples were measured in an Instron 5967 testing equipment (Instron, Norwood, MA, USA). Eight rectangles of 0.5 × 4.5 cm^2^ were cut from each sample and their elongation (E%), tensile strength (TS) and Young’s modulus (YM) was measured. The employed transverse test was 3 mm/min and the 500 N of load cell were used.

#### 2.6.10. Determination of the pH of the Point of Zero Charges (pH_PZC_)

The pH of the point of zero charges (pH_PZC_) of the PA-CMC adsorption membrane was measured by the pH drift technique, as Khadhri et al. [39] outlined. Firstly, a series of bottles containing 50 mL of NaCl aqueous solution (0.01 M) at pH ranging from 2 to 12 were prepared. Then, 0.2 g of the prepared adsorption membrane was added to each solution and nitrogen was bubbled inside each bottle in order to stabilize the pH by preventing the dissolution of the CO_2_. After stirring for 24 h at room temperature, the solid fraction was removed from the solution by filtration and the pH values of the filtrate were measured. The final pH (pH_f_) of the solution was measured and plotted against the initial (pH_i_) and by plotting pH_f_ vs. f(pH_i_), the pH_ZCN_ of the PA-CMC adsorption membrane was measured.

### 2.7. Adsorption Capacity Test of the PA-CMC Membrane

Using a Backman UV/Vis DU 800 spectrophotometer (Beckman Coulter, Inc., Fullerton, CA, USA), the amount of MG was measured. According to Equation (7), the equilibrium adsorbed quantity (Q_e_ (mg/g)) was calculated:

(7) $$Q_{e}=\frac{\left(\mathrm{Co}-\mathrm{Ce}\right)\;V}{m}$$

where C_0_ and C_e_ (mg L^−1^) represent the starting and final MG concentrations, V (L) represents the volume of the solution, and m (g) represents the mass of the PA-CMC adsorption membrane.

#### 2.7.1. Effect of Adsorption Parameters

To test the impact of the initial pH of the solution, 10 mL of 100 mg/L MG solution was mixed with 10 mg of adsorbent. The pH was changed to 2, 4, 6, 8, 10, or 12 and the various solutions were agitated for 720 min.

The same experiments were carried out to determine the adsorbent dose, except that the contact duration was set at 720 min and the adsorbent dose was adjusted to: 0.2, 0.4, 0.8, 1, 1.5, 2, and 3 g/L.

The same procedures were used to find the ideal contact time; 10 mg of adsorbent was stirred with 10 mL of the MG solution (50, 100, and 150 mg/L) (initial pH = 10) for the appropriate contact duration up to 720 min.

#### 2.7.2. Adsorption Equilibrium Study

Tests were carried out by adjusting the starting MG concentration (20–200 mg/L) (initial pH = 10) for contact times ranging from 30 to 720 min. Freundlich and Langmuir models were utilized to modulate experimental points and evaluated using the regression coefficient (R^2^), the chi-square test of nonlinear analysis (χ^2^), the residual root mean square error (RMSE), and the normalized standard deviation (ΔQ).

## 3. Results and Discussion

### 3.1. Influence of Extraction Process on the Obtained Cellulose Characteristics

The trunk of the *Ziziphus lotus* was subjected to two procedures: an alkaline process and a delignification-bleaching treatment (T 203 cm-99) [23] to extract its cellulosic fraction. During these two processes, the hemicelluloses and lignin fractions of the *Ziziphus lotus* are solubilized providing a solid residue mainly composed by cellulose [40,41]. When the *Ziziphus lotus* was subjected to an alkaline process 31.3% of the feedstock was solubilized, while when it was subjected to the TAPPI standard method a 35.8% was solubilized. To determine the characteristics of the isolated cellulosic fractions, the length of their fibers was determined, since the longer the cellulose fibers the more chances have the functional groups of the cellulose to interact with the reagent [42]. In this case, the longer fibers of the alkaline cellulose (PA-Cell) (737 vs. 689 µm of the α-Cell) allow for a higher conversion of the cellulose into carboxymetilcellulose (59.5 vs. 54.3% for the α-CMC).

### 3.2. Characteristics of the Obtained Carboxymethylcelluloses (PA-CMC vs. α-CMC)

The carboxymethylcelluloses produced from the isolated celluloses were deeply characterized to ascertain how the used extraction process influences their structures. They were subjected to XRD analyses and to degree of substitution and polymerization degree determinations in order to verify the formation of the carboxymethylcelluloses and to select the CMC that was employed for the production of the adsorption membranes.

#### 3.2.1. Determination of the Structure of the PA-CMC and α-CMC

The structure of the carboxymethylcelluloses is a crucial factor to employ them for wastewater remediation since it renders them soluble in water. During the carboxylation process, the crystalline structure of the cellulose is destructed by the swelling produced by sodium hydroxide before the addition of monochloroacetic acid (MAC) [43]. Thus, the PA-CM and α-CMC presented a lower fiber length than the cellulose from which they have been produced (354.6 and 320.9 µm, respectively vs. 776 µm of the PA-Cell and 689 µm of the α-Cell). Another, structural feature of the cellulose that is changed during the carboxylation process is the polymerization degree (DP), which must be below 0.4 in order to have a high hydroaffinity and a high solubility in water [44]. In this case, the PA-CMC was more soluble than α-CMC as they present a degree of substitution (DS) of 0.85 and 0.74, respectively. The higher the degree of substitution and the grafting rate, the lower is the polymerization degree, due to the degradation of the cellulose during the production of the CMCs [45,46]. According to this, the PA-CMC presented a lower polymerization degree than the α-CMC (320 and 470, respectively). Since the PA-CMC, due to its higher DP and lower DS, was more soluble in water than the α-CMC, it was the carboxymethylcelluloses used for the production of the adsorption membrane.

#### 3.2.2. XRD Analyses of the Produced Carboxymethylcelluloses

**Figure 1:**
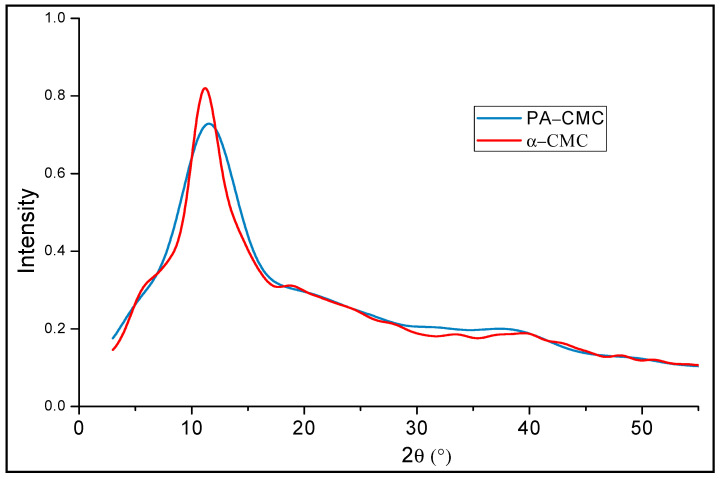
XRD spectra of the CMCs synthetized from the *Ziziphus lotus*.

To confirm that the carboxymethylation reaction of the alkaline and α-cellulose has occurred, the obtained PA-CMC and α-CMC were subjected to a XRD analyses. Commonly, the cellulose presents characteristics peaks at 2θ = 15.7°, 22.1° and 34.5°, which are associated to the (101), (002), and (400) lattice planes of cellulose [47]. However, the XRD spectra of the PA-CMC and α-CMC presented a single broad band at 2θ~18° and 2θ = 20°, respectively, as it can be seen in Figure 1. This band is associated with the amorphous phase of the cellulose, so it demonstrate that the ordered structure of the cellulose has been disrupted during the carboxymethylation of the cellulose, similar to what Gao et al. [47]. Mazuki et al. [48] suggested that during the etherification of the cellulose with MAC the hydrogen bonds of that join the cellulose chains are cleaved.

Although the XRD spectra of the obtained CMC present a very similar pattern, their crystallinity index (CrI) was different. The CrI of the PA-CMC was lower than the one of the α-CMC (53.31 vs. 59.47%), facilitating its dissolution in water, which is an essential requirement in order to be used in the production of the adsorption membrane in an aqueous media.

### 3.3. Formation and Characterization of the PA-CMC Adsorption Membrane

To determine how the structure of the cellulose extracted by an alkaline process has changed during all the reactions carried out to form the adsorption membrane the PA-Cellulose, PA-CMC and PA-CMC adsorption membrane were characterized by FTIR and SEM. Furthermore, in order to confirm the production of the cross-link between the citric acid and the PA-CMC, the PA-CMC and the formed adsorption membrane were subjected to water solubility tests. To determine the effect caused by citric acid, the mechanical properties of these samples and the water contact angle were determined.

#### 3.3.1. Changes in the Structure of the PA-Cellulose during the Formation of the PA-CMC Adsorption Membrane

To determine the changes that cellulose extracted from the *Ziziphus lotus* by an alkaline process has to suffer in order to form the PA-CMC adsorption membrane, the three samples (PA-Cell, PA-CMC and PA-CMC adsorption membrane) were analyzed by FTIR and SEM.

**Figure 2:**
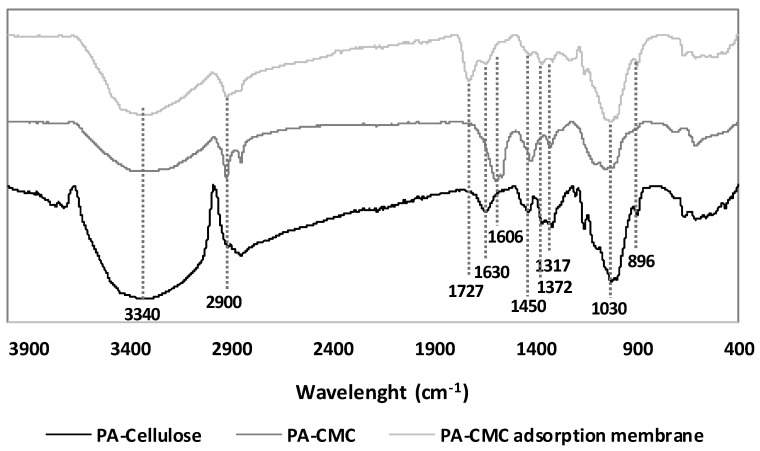
FTIR spectra of PA-celluloses, PA-CMC and the PA-CMC-adsorbent.

The FTIR spectra of these three samples are shown in Figure 2. It could be observed in this Figure that the FTIR spectra of the three samples present mainly the same bands, since the basic structure of the carboxymethylcellulose and of the adsorption membrane is cellulose.

The bands observed at 3340 and 2900 cm^−1^ correspond to the stretching frequency of hydroxyl groups in cellulose and to the stretching vibration of the C-H bond [47]. The bands observed at 1450, 1372 and 1317 cm^−1^ are attributed to the crystalline structure of the cellulose, while the band observed at 1030 cm^−1^ is characteristic of the cellulose [49]. The band observed at 896 cm^−1^ corresponds to the amorphous cellulose [50].

However, after the carboxymethyl reaction the fingerprint spectra of the cellulose suffer some changes, as it could be seem in the FTIR of the PA-CMC. It could be observed that the band that the cellulose presented at 1630 cm^−1^ to the bending vibration of the water absorbed by the cellulose has shifted to 1606 cm^−1^, which corresponded to the asymmetric tensile vibration of the COO- group [51]. It could also be appreciated that the band observed at 1450 cm^−1^ also shifted to 1420 cm^−1^, which corresponded to the symmetric tensile vibration of the COO- group [51]. Thus, these two new bands confirmed the formation of PA-CMC by the carboxymethylation of the cellulose.

Regarding the FTIR spectra of the PA-CMC adsorption membranes, it could be observed that it presented a spectra that resembles more the cellulose than the carboxymethylcelluloses but with a new band at 1727 cm^−1^. This new band indicates the formation of an ester bond between the hydroxyl groups of the CMCs and the anhydride formed from the citric acid, following the mechanism suggested by de Lima et al. [52].

**Figure 3:**
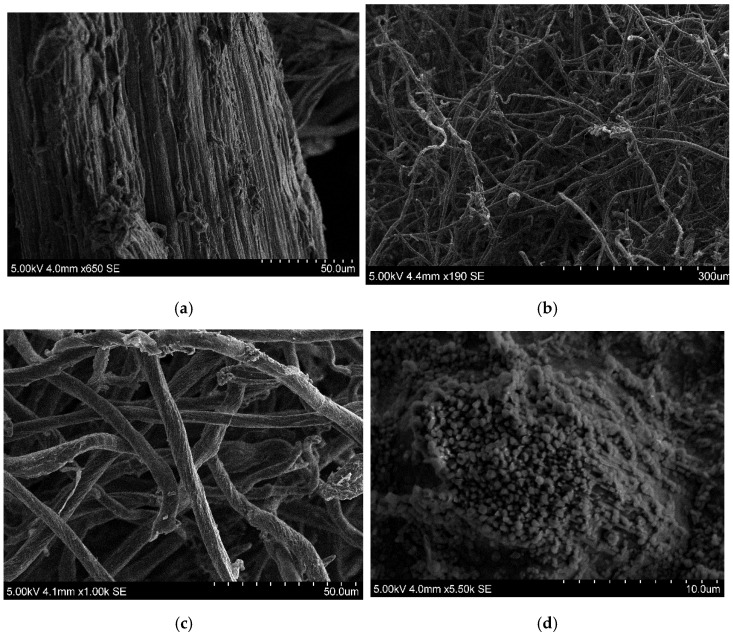
SEM images of the untreated *Ziziphus lotus* (**a**), PA-Cellulose (**b**), PA-CMC (**c**) and PA-CMC adsorption membrane (**d**).

Apart from the Fourier Transform Infrared (FTIR) spectroscopy, the changes that the structure of the cellulose obtained suffers during the different reactions was also visualized by SEM, as it could be seen in Figure 3. As it can be seen by comparing Figure 3a–c the surface structure of the untreated *Zizyphus lotus* and of the extracted alkaline cellulose were flat and stiff, whereas the PA-CMC presented rod and ribbon shapes [53]. Due to the destruction of the hydrogen bonds present in the cellulose by the alkalization and etherification reactions that take place during the production of the CMCs, their surface appeared rough with appreciable cracks and grooves than the ones of the corresponding celluloses, similar to what Chen et al. [54] observed.

The adsorption membrane prepared using PA-CMCs had a rough appearance with granular features and relief, together with a porous surface. The presence of these pores in the membrane may be due to the formation of a macromolecular network following the cross-linking reaction with citric acid. This could provide the PA-CMC adsorption membrane with suitable absorption capacities.

#### 3.3.2. Effect of the Addition of the Citric Acid in the Formation of the PA-CMC Adsorption Membrane

With the aim of confirming the crosslinking effect that citric acid had among PA-CMC chains, water solubility analyses were carried out. To do so, the amounts of PA-CMC and PA-CMC adsorption membrane that were dissolved in water at room temperature for 24 h were quantified. As expected, the amount of solubilized PA-CMC (approximately 85 ± 2.0%) was significantly higher than that found for PA-CMC adsorption membrane (27.5 ± 2.5%). In fact, visually, PA-CMC sample was almost completely dissolved during the first 2 h. Thus, these results clearly confirm the successful crosslinking reaction with citric acid, confirming what it was observed in the results of the FTIR analyses, Section 3.3.1 [28,55].

Since the PA-CMC was almost soluble in water, only the hydrophilicity of the synthesized PA-CMC adsorption membrane was evaluated by water contact angle analyses. It is well know that angles below 90° are indicative of the wettability of the analyzed surface, whereas the ones above 90° are characteristic of hydrophobic materials. The average initial contact angle for the adsorption membrane was of 68.4 ± 5.4°, meaning that the synthesized membrane was hydrophilic, which could be explained by the hydrophilic carboxyl and hydroxyl groups remaining on the surface of the PA-CMC adsorption membrane [5], as was observed in the FTIR spectra of this membrane in Section 3.3.1. In fact, after less than one minute, the water drop had completely disappeared in all the samples. However, it is worth to mention that none of the samples got holed, which was related to the crosslinking effect of citric acid. The reported water contact angle was higher than those reported by some authors for cellulose and lignocellulosic films [55,56,57]. Nevertheless, as reported by Shao et al., an increase in the amount of citric acid can lead to higher water contact angles in lignocellulosic films [55].

Regarding the mechanical properties of the carboxymethylcellulose and of the adsorption membrane, at first sight, PA-CMC seemed much more fragile than the membrane. This was confirmed by tensile tests, since the results for their Young’s Modulus were of approximately 883 ± 50 MPa and 3228 ± 530 MPa for PA-CMC and PA-CMC adsorption membrane, respectively. Consequently, both samples presented low elongation at break (<2%). This increase on the rigidity was directly related to the effect of citric acid as a crosslinker between PA-CMC chains, and was on the range of the values reported by Shao et al. [55] for lignocellulosic fibers crosslinked with this acid. Thus, the addition of citric acid clearly improved the mechanical properties of this material, leading to a resistant adsorption membrane.

### 3.4. Evaluation of the Capacity of the PA-CMC Adsorption Membrane to Adsorb Methyl Green

To evaluate the potential of the PA-CMC adsorption membrane in the wastewater remediation its capacity to adsorb the cationic dye methyl green (MG) was determined. To study in depth the adsorption process the influence of different parameters that could affect the capacities of the adsorption membrane, such as the pH, the dose and the contact time were evaluated. Furthermore, the kinetics and the isotherms of the adsorption process were studied.

#### 3.4.1. Effect of Different Adsorption Parameters

**Figure 4:**
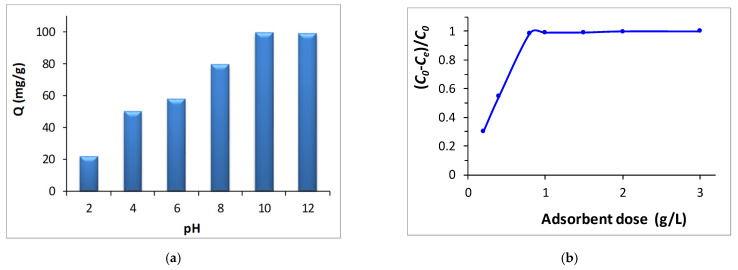

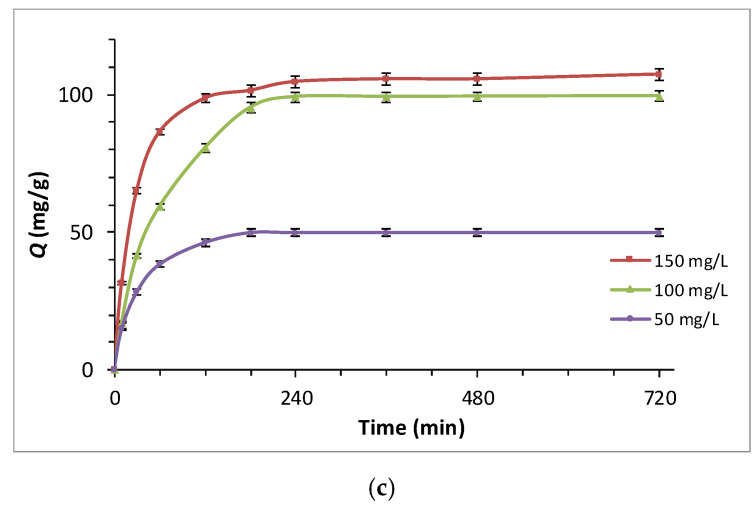
Influence of starting pH (**a**), adsorption membrane dosage (**b**) and contact time (**c**) on the adsorption of methyl green.

First of all the influence of the initial pH in the adsorption capacities of the membrane were evaluated. As it can be appreciated in Figure 4a, the maximum removal capacity of the dye was achieved at pH 10 (99.17 mg g^−1^). Once that solution’s pH exceeds the PA-CMC adsorption membrane’s pH_PZC_ (pH_PZC_ = 9.2), its surface is negatively charged, which favors the adsorption of the methyl green due to the electrostatic attraction that is generated between them. These electrostatic interactions will increase when the pH increases since the negative charged of the surface of the adsorption membrane will increase, similar to what Zhaozhao et al. [58] observed.

Another key factor that could influence the adsorption process is the adsorbent dose or the quantity of adsorption membrane that is employed since the number of adsorption sites would depend on this [59]. As it can be seen in Figure 4b the higher the employed dose the higher is the removal of the methyl green. The MG removal efficiency increases from 30% to 99% as the adsorption membrane dosage rises from 0.2 to 1 g/L. However, beyond 1 g/L dose, the MG removal remains unchanged. As a consequence, 1 g/L of adsorption membrane will be used to analyze adsorption isotherms.

Apart from the influence of the initial pH and the adsorption dosage, the impact of contact time on the rate of MG removal was assessed in order to establish the time needed for efficient MG removal. As shown in Figure 4c the removal rate of MG by the synthesized PA-CMC adsorption membrane increases when the contact time varies from 0 to 240 min. However, beyond 240 min the removal percentage of the MG remains unchanged. So, 240 min of contact time was deemed enough to reach the equilibrium.

Thus, it was observed that using an initial pH of 10, an adsorbent dosage of 1 g/L after 240 min the adsorption capacity (Q_m_) of the adsorption membrane was 121.5 mg/g. The ability of adsorbing methyl green of the adsorption membrane produced from the cellulosic fraction of the *Ziziphus lotus* was much higher than the one reported by other authors for adsorbents such as graphite oxide, loofah fibers or bamboo (29.4 mg/g, 18.2 mg/g and 15.5 mg/g, respectively) [60,61,62]. Nevertheless, there are adsorbents such as the graphene sheets and the sodium alginate hydrogel, which presented higher than that of PA-CMC adsorption membrane prepared from *Zizyphus lotus* (203.5 mg/g and 1915 mg/g respectively [60,63].

#### 3.4.2. Adsorption Kinetic Studies

To have a deeper understanding of the mechanism of the PA-CMC adsorption membrane to adsorb methyl green the use of the laws of pseudo-first-order (Equation (8)) and pseudo-second-order (Equation (9)) kinetics were evaluated [18,39,64].

(8) $$\ln\left(Q_{e}-Q_{t}\right)={\mathrm{lnQ}}_{e}-K_{1}t$$

(9) $$\frac{t}{Q_{t}}=\frac{1}{Q_{e}^{2}\times K_{2}}+\frac{t}{Q_{e}}$$

where Q_t_ (mg/g) is adsorption capacity at time “t” and K_1_ (min^−1^) and K_2_ (g/(mg min)) are the constants for pseudo-first-order and for pseudo-second-order, respectively.

The calculated MG adsorbed quantity values (Q_cal_) using the pseudo-second-order model, are comparable to the observed results (Q_exp_) (Table 1). In addition, the values of the regression coefficients in the case of pseudo-second order model are high compared to those found by the pseudo-first-order model (Table 1). Similarly, the pseudo-second-order model’s error functions are lower than pseudo-first-order’s. These results indicate that the MG adsorption on PA-CMC adsorption membrane follows a pseudo-second order kinetic model.

#### 3.4.3. Isotherms Study

To find the model that suits better the experimental results, two different models were evaluated; the Freundlich and Langmuir models [65]. The results of modeling the experimental data are shown in Table 2.

According to the results shown in Table 2, the Langmuir model was determined to be the best suitable (R^2^ = 0.969) to explain the adsorption of MG on PA-CMC adsorption membrane. Moreover, this result was verified by the low values of χ^2^, ∆q and RMSE, which reflects that the monolayer adsorption with weak interaction of the adsorbate on energetically identical surface. Therefore, it could be concluded that MG was adsorbed chemically on energetically identical sites and with the formation of a molecular monolayer [64].

## 4. Conclusions

This study presents the synthesis of CMC obtained from the *Ziziphus lotus* as a biorefinery route for the revalorization of this undervalued feedstock. The obtained CMCs were comprehensively characterized and the most soluble CMC was used for the production of an adsorption membrane. Between the PA-CMC and the α-CMC, it was observed that the first one presented a higher degree of substitution and a lower crystallinity than the α-CMC (53.31 vs. 59.47%), which confers a higher solubility. Since the solubility of the CMC is a crucial parameter to form the adsorption membrane, PA-CMC was the carboxymethylcellulose cross-linked with citric acid. After confirming the formation of the cross-link of the PA-CMC with citric acid, it was observed that this acid confers hydrophobicity and rigidity to the membrane. Furthermore, the resistant membrane produced from the PA-CMC extracted from the Ziziphus lotus presented a higher contact angle than other cellulose or lignocellulosic films. The capacity of the produced membrane to adsorb a cationic dye such as methyl green was evaluated, being observed that its adsorption capacity was 121.5 mg/g, which is higher than the capacity reported for other adsorbents. It was also observed that the adsorption of the methyl green by the produced adsorption membrane follows the Langmuir model and the pseudo-second-order model, which implies that the adsorption is a monolayer phenomenon that most likely takes place on sites that are energetically uniform. Thus, this work proposes the production of a membrane suitable for water remediation from an unexploded feedstock by green processing.

## Figures and Tables

**Table 1:** Kinetics parameters for the MG adsorption.

**[MG] (mg L^−1^)**	**Q_exp_** **(mg g^−1^)**	**Pseudo-First-Order**					
		**Q_cal_** **(mg g^−1^)**	**K_1_** **(min^−1^)**	**R^2^**	**χ^2^**	**∆Q**	**RMSE**
50	49.96	16.17	0.0160	0.07740	377.097	0.3968	27.614
100	99.52	68.72	0.0144	0.8779	103.322	1.3619	29.792
150	107.4	37.29	0.0080	0.8458	671.176	0.3686	55.938
		**Pseudo-Second-Order**					
		**Q_cal_** **(mg g^−1^)**	**K_2_** **(g mg^−1^ min^−1^)**	**R^2^**	**χ^2^**	**∆Q**	**RMSE**
50	49.96	51.54	0.0011	0.9991	40.77	0.1013	15.291
100	99.52	106.38	0.0002	0.9997	69.034	0.3984	21.362
150	107.4	109.89	0.0005	0.9997	81.336	0.0707	31.524

**Table 2:** MG adsorption isotherms constants.

**Langmuir**	
Q_m_ (mg/g)	121.58
K_L_ (L/g)	0.0216
R_L_	0.0029
R^2^	0.969
χ^2^	5.63
Δq	0.0088
RMSE	7.81
**Freundlich**	
1/n	1.583
K_f_ (L/g)	0.742
R^2^	0.809
χ^2^	56.65
Δq	0.1419
RMSE	22.66

## Data Availability

Not applicable.
